# International Medical Annual

**Published:** 1889-05

**Authors:** 


The International Medical Annual and Practitioners' Index for 1889. Price, $2.75; pages 580. E. B. Treat & Co., New York and London.

Medical annuals have of late years become a prominent feature in medicine. The *International Annual* gives the latest ideas and theories current in medicine, surgery, and therapeutics; thus, whatever there may be that is valuable is brought within ready reference for the busy practitioner. All reference to homœopathic literature is unfortunately omitted.

